# EEG-MEG Integration Enhances the Characterization of Functional and Effective Connectivity in the Resting State Network

**DOI:** 10.1371/journal.pone.0140832

**Published:** 2015-10-28

**Authors:** Muthuraman Muthuraman, Vera Moliadze, Kidist Gebremariam Mideksa, Abdul Rauf Anwar, Ulrich Stephani, Günther Deuschl, Christine M. Freitag, Michael Siniatchkin

**Affiliations:** 1 Department of Neurology, Christian-Albrechts-University, Kiel, Germany; 2 Institute of Medical Psychology and Medical Sociology, Christian-Albrechts-University, Kiel, Germany; 3 Department of Child and Adolescent Psychiatry, Psychosomatics and Psychotherapy, Goethe-University Frankfurt am Main, Frankfurt, Germany; 4 Digital signal processing and system theory, Christian-Albrechts-University, Kiel, Germany; 5 Department of Neuropediatrics, Christian-Albrechts-University, Kiel, Germany; University of Rome, ITALY

## Abstract

At the sensor level many aspects, such as spectral power, functional and effective connectivity as well as relative-power-ratio ratio (RPR) and spatial resolution have been comprehensively investigated through both electroencephalography (EEG) and magnetoencephalography (MEG). Despite this, differences between both modalities have not yet been systematically studied by direct comparison. It remains an open question as to whether the integration of EEG and MEG data would improve the information obtained from the above mentioned parameters. Here, EEG (64-channel system) and MEG (275 sensor system) were recorded simultaneously in conditions with eyes open (EO) and eyes closed (EC) in 29 healthy adults. Spectral power, functional and effective connectivity, RPR, and spatial resolution were analyzed at five different frequency bands (delta, theta, alpha, beta and gamma). Networks of functional and effective connectivity were described using a spatial filter approach called the dynamic imaging of coherent sources (DICS) followed by the renormalized partial directed coherence (RPDC). Absolute mean power at the sensor level was significantly higher in EEG than in MEG data in both EO and EC conditions. At the source level, there was a trend towards a better performance of the combined EEG+MEG analysis compared with separate EEG or MEG analyses for the source mean power, functional correlation, effective connectivity for both EO and EC. The network of coherent sources and the spatial resolution were similar for both the EEG and MEG data if they were analyzed separately. Results indicate that the combined approach has several advantages over the separate analyses of both EEG and MEG. Moreover, by a direct comparison of EEG and MEG, EEG was characterized by significantly higher values in all measured parameters in both sensor and source level. All the above conclusions are specific to the resting state task and the specific analysis used in this study to have general conclusion multi-center studies would be helpful.

## Introduction

Electroencephalography (EEG) and magnetoencephalography (MEG) represent functional imaging techniques which measure neural activity with a high temporal resolution. However, each of these methods is characterized by specific advantages and drawbacks. MEG is superior to EEG for identifying brain sources with tangentially oriented dipoles and in addition, captures short range connectivity better [1]. In contrast, EEG is characterized by more precise localization of brain sources with radially oriented dipoles, and better describes long range connectivity [2]. Finally, EEG is more prone to volume conduction effects than MEG [3]. Still, not all functional quantities that can be derived from estimated brain activity have been compared between EEG and MEG. Spectral power, functional and effective connectivity as well as relative-power-ratio (RPR) and spatial resolution have been sufficiently investigated on the sensor level using both EEG and MEG. Despite this, a direct comparison of the differences between these modalities has yet to be systematically performed. Moreover, the disadvantages of EEG and MEG may be compensated for by utilizing a combination of both modalities, as demonstrated in a number of studies [4–15]. To the best of our knowledge, no previous studies have shown whether the integration of EEG and MEG data can improve the analysis of spectral power, functional and effective connectivity, and RPR. Here, we first compare the sensitivity of EEG, MEG, and the combined EEG-MEG analysis for the detection of the parameters listed above. We use the resting state EEG and MEG data with two conditions, eyes closed (EC) and eyes open (EO), because of well described oscillatory networks underlying these resting state networks (see Michels et al., 2013). Previous studies have shown differences between EC and EO conditions based on the power spectrum [16, 17], but only a few have looked at differences in functional brain networks between EC and EO conditions [18–21]. The second aim of this study, therefore, is to compare EEG and MEG derived measurements in relation to EC and EO conditions.

Dynamic imaging of coherent sources (DICS) is one of the source analysis techniques in the frequency domain. DICS uses adaptive spatial filtering to analyze neuronal networks by imaging power and coherence estimates of oscillatory brain activity [22]. There is increasing evidence that DICS is able to show coherent changes not only between cortical sources, but also between cortical and sub-cortical structures such as the thalamus [15, 23–32]. The delineation of neuronal activity requires not only the information on localization, but also on the interactions between the activated sources. Using the renormalized partial directed coherence (RPDC), directed connectivity can be investigated by accessing the information flow between different sources. The combination of both these approaches allow analyses of the functional (coherences between cortical and sub-cortical activities) and effective (hierarchical relation and information flow between involved regions) connectivity within neuronal networks.

In this study, we investigate detection sensitivity of EEG, MEG as well as of EEG-MEG fusion, for the analysis of spectral power, functional and effective connectivity, and RPR in conditions EO and EC using both DICS and RPDC.

## Subjects and Methods

### 2.1 Subjects

Twenty nine (15 male and 14 female) healthy volunteers participated in this study (age 29 ± 5,68 years, age range: 23–38 years). The subjects were recruited by advertisements. The study was performed in accordance with the Declaration of Helsinki and approved by local ethics committees of the Medical Faculty, Goethe Universität Frankfurt am Main. All subjects gave their written informed consent. The subjects were paid for participation in this study.

### 2.2 Experimental design

First, each subject maintained an eyes-closed state for five minutes in a fully relaxed state. Second, the subjects kept their eyes-open for another five minutes while focusing on a cross in the middle of a screen without any instructions and staying in a fully relaxed state. MEG measurements were recorded using a 275 channel whole-head CTF MEG System (Omega 2005; VSM MedTech Ltd.) in a synthetic 3rd-order gradiometer configuration. EEG measurements were obtained simultaneously using CTF EEG amplifiers integrated with the MEG system. 56 channels selected from EEG caps with 61 equidistantly placed scalp electrodes (64-channel system, BrainProducts, München, Germany) were used for EEG recordings. EEG and MEG measurements were synchronously recorded at a sampling rate of 1200 Hz and filtered online with fourth-order Butterworth filters (300 Hz low pass and 0.1 Hz high pass). Before and after each five minute recording the subject's head position relative to the gradiometer array were determined using three localization coils, one at the nasion and the other two located 1 cm anterior to the tragus of each ear on the nasion-tragus plane. Epochs with a head movement exceeding 5 mm were discarded from further MEG/EEG data analysis. For artefact detection a horizontal and vertical electro-oculogram (EOG) was recorded via four electrodes; two were placed distal to the outer canthi of the left and right eye (horizontal eye movements) and the other two were placed above and below the right eye (vertical eye movements and blinks). The impedance of each electrode was measured with an electrode impedance meter (Astro-Med, Inc Grass Instrument Division, W. Warwick RI USA) and was kept below 15 kΩ. Data were stored in a computer and analyzed off-line.

### 2.3 Data pre-processing

Each recording was segmented into a number L of 1s - long high-quality epochs, discarding all those data sections with visible artifacts. The total length (N) of the recording was divided into M high quality segments with M ranging from 280 to 290, such that N = LM. The data were analyzed across the following frequency bands: delta (1–3 Hz), theta (4–7 Hz), alpha (8–13 Hz), beta (14–30 Hz) and gamma (31–49 Hz) for each condition of EO and EC separately.

### 2.4 Semi-realistic head models

The solution for the forward problem in this study is achieved by an approach which uses the piece-wise homogeneous approximation and can be estimated by boundary element method (BEM) [12, 33–38]. The conductivity of the BEM model is assumed to be isotropic for each layer of the head. The lead field matrix relates the current sources present within the brain to the electromagnetic activity measured across the scalp. The vector lead field matrix estimated here contains the information regarding the geometry and conductivity of the model. The standard magnetic resonance images (MRI) are used for extracting the surfaces of the layers such as the scalp, skull, and brain. The individual electrodes and sensors locations were obtained from the CTF system. The linear-collocation 3-layer BEM model was used to construct the semi realistic head models. The open source software OpenMEEG [39] was used to construct the semi-realistic head models. The backbone of this approach was developed on the basis of the integrated analysis of MEG and EEG simultaneously. The conductivity is a minor concern in the case of MEG[34], is used first to find the accurate source location information for the tangential components. Next the radial component from the EEG data by adjusting the conductivity profile of the EEG model in integrated [12]. The conductivity values for the scalp and brain was 0.8 S/m, and for the skull 0.008 S/m. The number of elements of the BEM was 3564 for the grey matter and the 8723 covering the entire brain with a 5 mm voxel distance for each element.

### 2.5 Source analysis

The DICS beamformer has several advantages in comparison to other beamformer source analysis in the frequency domain. First, our primary objective was to look at the network of coherent sources to achieve this dynamic imaging of coherent sources is the most suitable method. The second was to use a beamformer which can be applied in the frequency domain so that we can look at the networks separately at each frequency band. Thirdly, EEG/MEG analyses such as DICS are based on a better temporal resolution; it is possible to apply calculations within the scope of Granger causality to demonstrate directionality of information flow within networks of sources by extracting the frequency band specific network source signals. The inverse problem is solved in this study using a beamformer approach called the dynamic imaging of coherent sources (DICS) [22] to identify coherent brain sources at predefined frequency bands. DICS uses a algorithm called the linear constrained minimum variance (LCMV) spatial filter [40] and estimates the tomographic power maps which are based on the semi-realistic head models as used in this study. The power and coherence at any given location in the brain can be estimated using a linear transformation which in this study is the LCMV filter. The filter relates the electromagnetic filed on the surface to the underlying neural activity in a certain brain region. The neural activity is modelled as a current dipole or sum of current dipoles. The spatial filter was applied to a large number of voxels covering the entire brain, assigning to each voxel a specific value of coherence. A voxel size of 5 mm was used in this study. The reference region was identified by the source in the brain with the strongest power first and used as the reference to find other coherent sources at the respective frequency bands. Once coherent brain areas were identified, their activity was extracted by the spatial filter [40]. The application of the spatial filter has been previously described [41]. The criteria used to identify areas was performed using a within subject surrogate analysis. This was used to define the significance level and set the limit for projecting out and identifying other areas in the brain. Local maxima in the resulting maps represent areas that have the strongest coherence to the reference signal. All the original source signals for each source with several activated voxels were combined (by estimating the second order spectra and employing a weighting scheme depending on the analyzed frequency range) to form a pooled source signal estimate for every source as previously described [42, 43]. This analysis was performed for each subject separately, followed by a grand average across all subjects for all the three recording methods EEG, MEG and the combined EEG-MEG approach (EEG+MEG).

### 2.6 Renormalized partial directed coherence (RPDC)

The RPDC was applied to identify the direction of information flow between two signals. [44]. For RPDC, the multivariate model is based on the principle of Granger causality [45]. The RPDC was used instead of Granger causality measures because RPDC provides both frequency information and causality information. The multivariate approach was used to model the pooled source signals as an autoregressive process to obtain the coefficients of the causality. The Akaike information criterion (AIC) was used in this study to estimate the optimal model order [46, 47]. The AIC is a measure of the relative goodness of fit which has the minimum loss of information of a resulting statistical model with an optimal order for the corresponding model [47]. The surrogates [48] was used to calculate the significance level on the pooled source signals after the estimation of the RPDC values. In the surrogate method [48, 49], we divide the original time series into smaller one second non-overlapping segments of equal size. These smaller one second windows are shuffled randomly and concatenated. This process was repeated 100 times and the 99^th^ percentile was taken as the threshold or significance value. In addition, the significant connections were tested with a time reversal technique for EEG to justify that observed connections are due to strong symmetries and not due to volume conduction effects in EEG [50, 51]. The open source Matlab package ARFIT [52, 53] was used for estimating the autoregressive coefficients from the spatially filtered source signals.

### 2.7 Relative-power-ratio (RPR) analysis

The scalp level RPR was estimated for both the recording modalities separately from the power spectrum of each of the electrodes/sensors. The RPR was calculated by taking the ratio of EC power (for each of the five individual frequency bands) to that of the power in the corresponding five individual frequency bands at the EO condition. The same numbers of sensors were selected from MEG and corresponding EEG electrodes to have a direct comparison of the RPR values. In total, minimum of 7 and maximum of 10 electrodes/sensors were selected with the maximum power at the particular frequency band for each of the five topological regions (frontal—9, central—7, parietal—9, temporal—10 and occipital—8) in the scalp. The selection of the electrodes and sensors was performed by estimating the Euclidean distance between the EEG electrodes and the corresponding MEG sensors (selected by visualization in the forward model). Our criteria stipulated that the Euclidean distance between the EEG electrodes and corresponding MEG sensors should be ≤ 20 mm. At the end, the mean RPR was estimated from the corresponding electrodes/sensors in case of MEG and EEG alone for the five topological regions separately. The source level RPR was estimated in the same way by taking the pooled source signals from the first identified sources in each recording modality separately for all frequency bands, instead of the electrodes/sensors signals. In case of EEG+MEG, the RPR was calculated by normalizing the source signals to the maximum RPR at the respective frequency band, yielding unit-free measures for both EEG and MEG [54].

### 2.8 Statistical analysis

At the scalp level, in a first step, the total data length between the subjects was tested with a non-parametric Friedman test for dependent samples (n = 29, α = 0,05). In a second step, in order to ensure that any of the reported results (which are all calculated for pre-defined frequency bands) are not confounded by group differences in individuals’ alpha frequency (IAF), we also estimated and compared individual band limits calculated as a percentage of the IAF [55]. First, we calculated the IAF from the mean of all EEG / MEG channels or sensors (excluding EOG and ECG channels). Next, based on the IAFs, we defined the lower and upper boundaries of the other frequency bands (delta, theta, and beta) within 10% of the predefined band edges. The mean and standard deviation of IAF from all the subjects was (10.4±2.3) Hz. For example, one subject had an IAF of 10,1 Hz, so the lower band edge for the delta band (defined as 1–3 Hz) is 1.01 Hz (0,1 (10% of 1 Hz) x 10,1 Hz) and the upper edge is 3.03 (0,3 x 10,1 Hz). We then estimated the median frequency band values for all subjects to see whether those values lay in the range of the pre-defined frequency band, and whether the values differed within the group of subjects (one-sample t-tests) (α = 0,05). In a third step, spectral source mean power differences within the different frequency bands of interest were assessed by two-tailed paired t-tests (α = 0,05). The significance threshold of the sources was tested by a within subject surrogate analysis. The surrogates were estimated by a Monte Carlo random permutation 100 times shuffling of one second segments within each subject. The one second epoch length was chosen by an adaptive epoch length selection method [56]. The p-value was estimated for each of these 100 random permutations and the 99^th^ percentile value of each source of all these permutations is taken as the final threshold.

At the source level, in a first step, the mean coherence (or interaction strength) between all sources was estimated. The Kruskal-Wallis one-way analysis of variance test was performed on the mean coherence values of EEG, MEG and EEG+MEG. In order to find the difference in coherence strength between the EC *versus* EO within recording modalities, a Friedman one-way analysis of variance test was performed with the coherence values on the first coherent source from each subject. In a second step, power and coherence differences between-recording modalities were assessed as follows: A reference voxel was selected in the posterior parietal cortex with the MNI co-ordinates [8–77 38]. The criteria for selecting this voxel were (1) highest frequency (number of occurrences) of activation in the identified first source in the network for all the frequency bands at the group level; (2) the voxel is found in the first identified source over all conditions (EO, EC); (3) it shows the lowest power of all voxels in all frequency bands, because the voxel to be selected here as the maximum power then it will overlap to the actual maximum power voxel. At the end, if the voxel has maximum coherence then this will also influence statistics for the RPDC analyses. The semi-realistic head model was used for all the recording methods which give the advantage of selecting the same reference voxel across the different recording methods. Within this spatial template, the Euclidean distance was estimated between the reference voxel and the voxel with the maximum power or coherence for the maximal overlapping (frequency of occurrence for each frequency band separately) number of sources between the recording modalities for all sources. The quantitative measure of distance was then compared between the recording modalities for each source with a one-way ANOVA. If the frequency band specific spatial distribution of sources differs within the recording modalities then there should be also significant difference between the recording modalities. This should be reflected by significant between recording method-differences in the Euclidean distances between the reference voxel and the particular overlapping sources.

In a third step, spatial differences between recording modalities were assessed as follows: the number of voxels activated for each source and each recording method (EEG, MEG, and EEG+MEG) was estimated. This quantitative measure was then compared between the recording modalities for each source with a one-way ANOVA. If the frequency band specific number of voxels differs within the recording modalities then there should also be significant difference between the recording modalities.

In a fourth step, to compare RPDC values between recording modalities analysis, the same reference voxel was chosen as described above. Next, the directionality or information flow was estimated between the reference voxel and the maximally activated voxel of all sources. The RPDC values were then compared between the three recording modalities with one-way ANOVA. The directionality between the reference voxel and the maximally activated voxel for each source is different between the three recording modalities. Since the directionality can be bi-directional (i.e., from the reference voxel to the maximally activated voxel and vice versa), we will report the subsequent results for both possible directions.

In a fifth step, the scalp level RPR between the recording modalities (EEG vs. MEG) was tested with a non-parametric Friedman test for dependent samples (n = 29, α = 0,05). The source signal RPR values (n = 29, α = 0,05) for each of the recording modalities were tested for significance using the multifactorial ANOVA, within-subject factor being the sources for EC (n = 4 sources: Delta;), (n = 3 sources: Theta), (n = 5 sources: Alpha), (n = 6 sources: Beta), for EO (n = 4 sources: Delta;), (n = 3 sources: Theta), (n = 5 sources: Alpha), (n = 4 sources: Beta) and the between subject factor being the recording modalities (n = 3: EEG, MEG, EEG+MEG). All the above mentioned statistical analyses were performed using Matlab R 2013 a- 64-bit version.

### 2.9 Simulation

In this study, we wanted not only to test the analyses on the real data as there are always several unknowns. We also wanted to test the analyses on data where we know the ground truth by simulations. The simulations of this kind can also help in building up model based studies is EEG and MEG for functional and effective connectivity. The two main purposes of the simulation are first to test the exact same analysis and the parameters estimated from real data to validate them with known values. The second to prove whether the RPR is an important parameter for the source analysis in comparison to the other factors like number of electrodes/sensors, orientation of the dipoles and the distribution of the sources.

Simulation1: Eyes closed

$$\begin{array}{l} x_{1}(t)=1,9775\;x_{1}(t-1)-0,9801\;x_{1}(t-2)+0,75\;x_{2}(t-1)+0,75\;x_{5}(t-1)+\eta_{1}(t)\\ x_{2}(t)=1,9775\;x_{2}(t-1)-0,9801\;x_{2}(t-2)+0,75\;x_{5}(t-1)+\eta_{2}(t)\\ x_{3}(t)=1,9775\;x_{3}(t-1)-0,9801\;x_{3}(t-2)+0,75\;x_{4}(t-1)+0,75\;x_{5}(t-1)+\eta_{3}(t)\\ x_{4}(t)=1,9775\;x_{4}(t-1)-0,9801\;x_{4}(t-2)+0,75\;x_{1}(t-1)+0,75\;x_{2}(t-1)+0,75\;x_{5}(t-1)+\eta_{4}(t)\\ x_{5}(t)=1,9775\;x_{5}(t-1)-0,9801\;x_{5}(t-2)+\eta_{5}(t) \end{array}$$ (1)

Simulation II: Eyes open

$$\begin{array}{l} x_{1}(t)=1,9775\;x_{1}(t-1)-0,9801\;x_{1}(t-2)+0,75\;x_{5}(t-1)+\eta_{1}(t)\\ x_{2}(t)=1,9775\;x_{2}(t-1)-0,9801\;x_{2}(t-2)+0,75\;x_{5}(t-1)+\eta_{2}(t)\\ x_{3}(t)=1,9775\;x_{3}(t-1)-0,9801\;x_{3}(t-2)+0,75\;x_{4}(t-1)+0,75\;x_{5}(t-1)+\eta_{3}(t)\\ x_{4}(t)=1,9775\;x_{4}(t-1)-0,9801\;x_{4}(t-2)+0,75\;x_{2}(t-1)+0,75\;x_{5}(t-1)+\eta_{4}(t)\\ x_{5}(t)=1,9775\;x_{5}(t-1)-0,9801\;x_{5}(t-2)+\eta_{5}(t) \end{array}$$ (2)

The source signals were modeled by auto regressive second order processes with independent noises. The noise *η*(*t*) was white Gaussian random noise with zero mean and unit variance. The signals were modeled with AR coefficients yielding a peak at 8 Hz in the frequency spectrum. In total six simulations were performed, three for both EC and EO conditions. For EC the simulations were performed separately for each modality (EEG, MEG and EEG+MEG) and then repeated for the EO. In all the simulations 360,000 data points have been simulated with a sampling frequency of 1,200 Hz. The simulation was repeated for 100 realizations. The reported results from the simulation are the mean of all the 100 realizations. The fitted model system a vector auto regressive process of model order 20 was used, thus, a considerably over fitting of the true model order which is of order 2. All the directed interactions simulated in the six simulations are taken from the real source analysis connectivity data from the healthy subjects in the alpha frequency band. The direct connectivity is guaranteed by non-zero coefficients in the model system.

The source dipoles were simulated with both radial and tangential orientations for all the three modalities namely EEG, MEG and the combined approach. The co-ordinates of the dipoles were derived by taking a rectangular grid of current dipoles which are placed on three-dimensional voxels. The voxel co-ordinates were derived by considering a rectangular grid of 27×23×27 voxels with distance 5 mm. An average human brain was then laid over these voxels and the 8723 voxels which were covering this brain were marked. Out of these voxels, the 3564 voxels which cover the grey matter of the brain were taken as the voxels used in the model. The brain model was derived from the average probabilistic MRI atlas from the Montreal neurological institute [57]. The source signals from these dipoles were inserted in neighboring voxels (n = 10 voxels for EEG; n = 6 voxels for MEG; n = 4 voxels for EEG+MEG) to create a current distribution instead of single voxel activation in all the modalities and conditions (in order to simulate more closely to the resting state data). The AR processes of order two as the generating source in the active voxels for each corresponding modality and additionally a propagation of the source into the inactive voxels by neighbor-interaction and additionally self-interaction. In detail, the following equation for the time series in the inactive voxels

$$\vec{j}\;(\upsilon ,t)=a_{i}\cdot \;\vec{j}\;(\upsilon ,t_{i}-1)+b_{i}\;\sum_{\upsilon^{\prime }\in \;N(\upsilon )}\vec{j}\;(\upsilon^{\prime }\;,t_{i}-1)\;i=1,2;$$ (3)

where *N*(*υ*) is the set of neighboring voxels of *υ*. The strength of the self-interaction and the neighbor interaction are defined by the parameters *a* and *b* respectively. While the order of the AR processes is given by *i* in the above Eq (3).

In all the three modalities EEG, MEG and EEG+MEG both the radial and tangential dipoles were simulated for the source signals as shown in S1 Fig The MNI location of the source dipoles were obtained from the source analysis grand averaged healthy subject’s data by taking the maximum voxel in each source and used for the simulation. The dipoles were simulated on both hemispheres for all the sources found in the alpha frequency band for EC and EO condition.

The difference in connectivity strength between the sources was tuned with the help of the independent noises *η*(*t*) in each source signal. In order to estimate RPR, the signal was defined as the power at a peak frequency of 8 Hz (EC), while the noise was defined as the power at 8 Hz (EO). In order to simulate the data closer to the real data the RPR was estimated from the grand average of the pooled source signals and implemented in the simulation. The RPR of the different sources for each of the modalities is given in S1 Table.

## Results

### 3.1 Analysis of spectral and source absolute mean power

**Fig 1:**
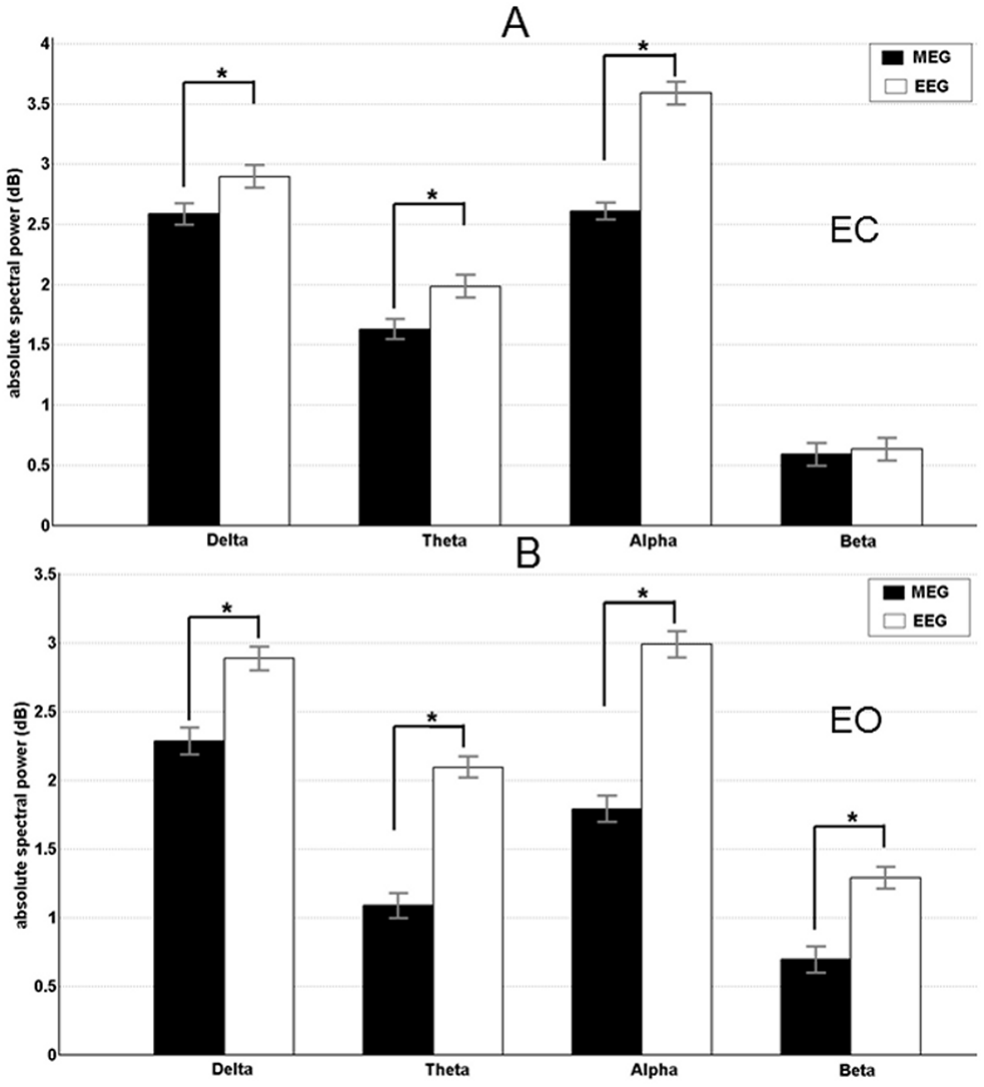
Results of the spectral absolute power for eyes open and eyes closed condition. Mean band power (with standard deviation) is shown for EEG (black bars) and MEG (white bars). A) Eyes open condition. B) Eyes closed condition. Significant recording method differences are indicated by * (p < 0.05).

**Fig 2:**
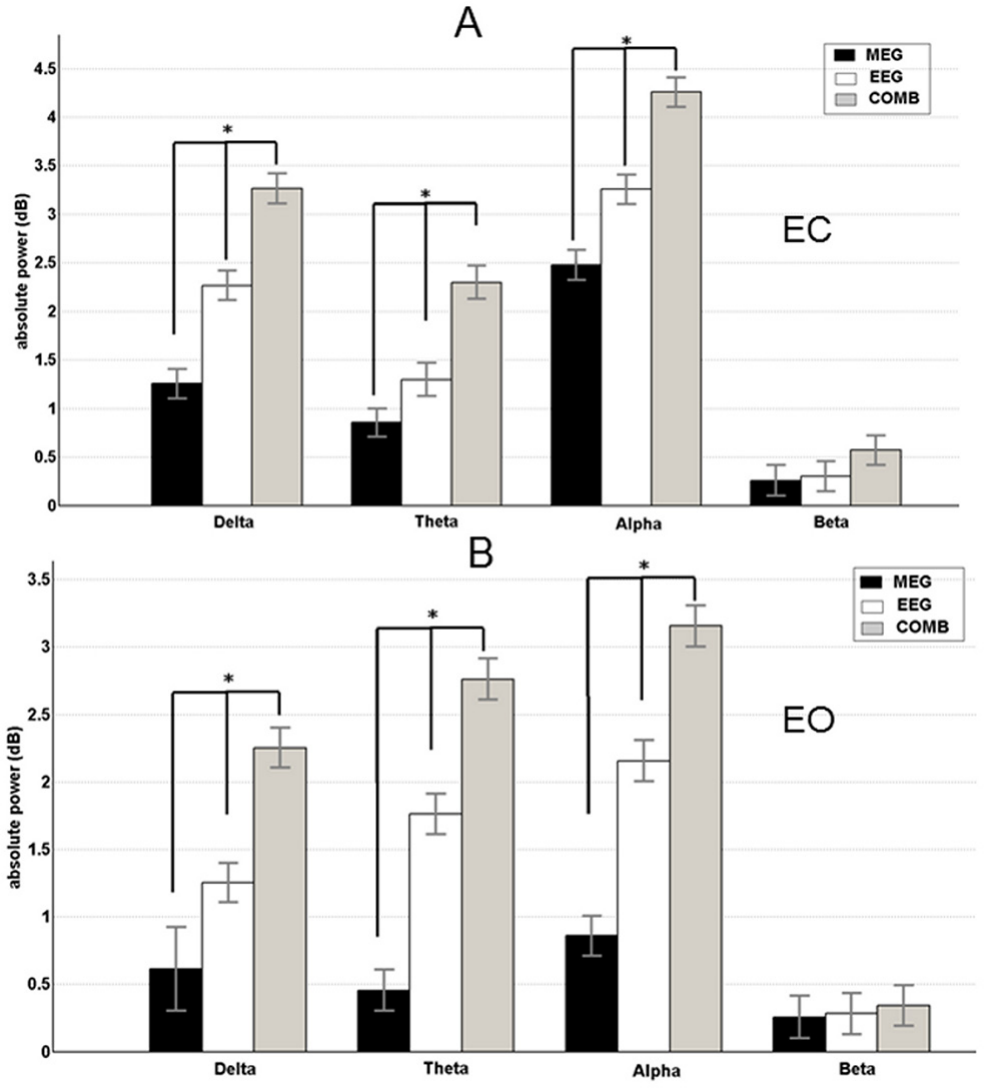
Results of the spectral source absolute power for eyes open and eyes closed condition. Mean band power (with standard deviation) is shown for EEG (black bars), MEG (white bars), and EEG+MEG (grey bars). A) Eyes open condition. B) Eyes closed condition. Significant recording method differences are indicated by * (p < 0.05).

The total data length was not different within the subjects (F_1,29_ = 1,86; p = 0,76). The IAF was not different between the subjects (one-sample t-tests), and the mean and standard deviation for all frequency bands were not different (S2 Table). Based on these findings, all results are reported according to the pre-defined frequency bands. All analysed bands showed significantly higher source and spectral absolute mean power for the EC compared to the EO condition in both recording modalities, EEG and MEG. The bar graphs of global spectral mean power differences between EEG and MEG are shown in Fig 1 and the source mean power is shown in Fig 2. In a comparison between the recording modalities for the spectral absolute mean power, values in the EEG data was significantly higher (S3 Table) than in MEG data across all the frequency bands for the EC condition. In the EO condition the same trend was observed across all except the beta frequency band (Fig 1). In the combined approach, the source absolute mean power was significantly higher (S4 Table) compared to the separate EEG and MEG analyses in all frequency bands except the beta band. Although not significant, it should be noted that the beta and gamma band (S6 Fig) data exhibited the same behaviour as the other frequency bands for both EC and EO conditions. Between EEG and MEG, EEG data showed significantly higher (S5 Table) source absolute mean power than MEG (Fig 2).

### 3.2 Coherent network of sources

**Fig 3:**
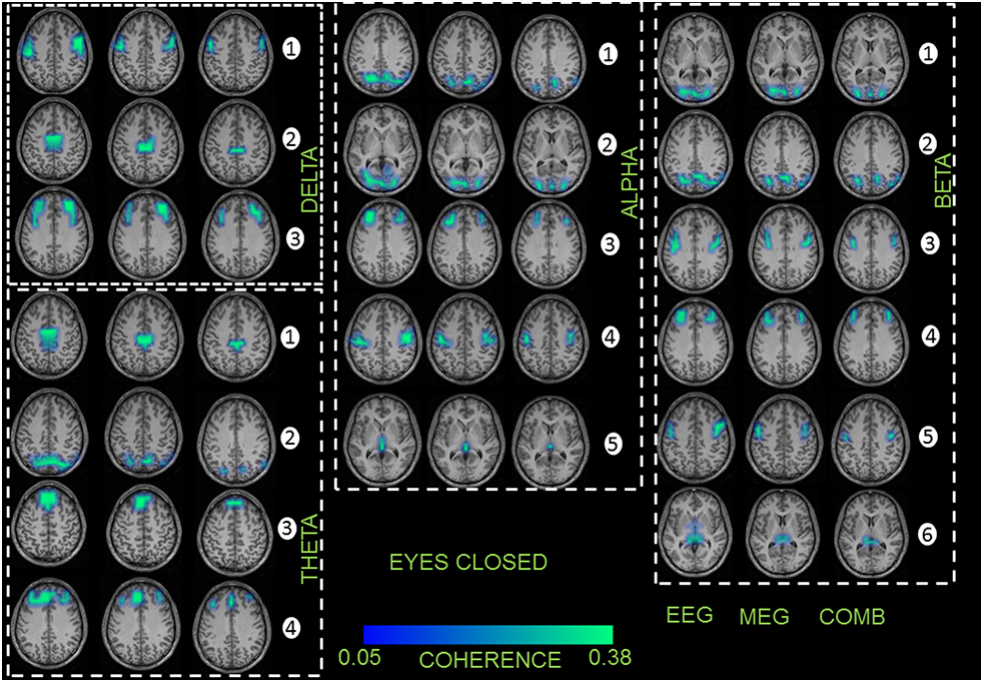
First column represents the recording method EEG in each frequency band showing the grand average statistical map of network of sources for the eyes closed (EC) condition. Second column represents the recording method MEG for each frequency band separately. Third column represents the combined approach (EEG+MEG). The numbers indicate the order of sources found for each frequency band separately.

**Fig 4:**
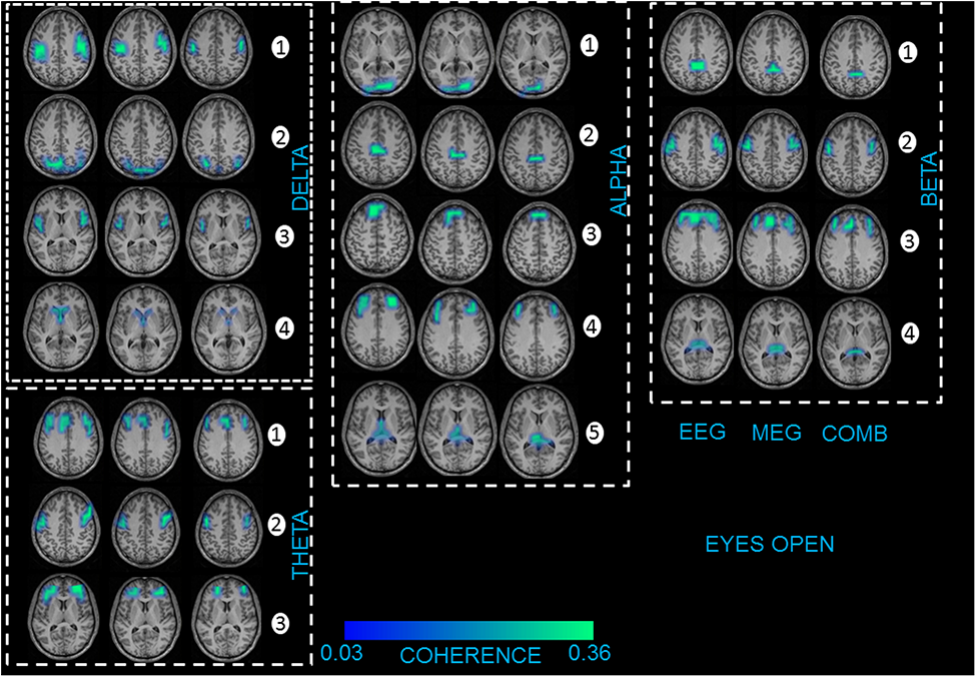
First column represents the recording method EEG in each frequency band showing the grand average statistical map of network of sources for the eyes open (EO) condition. Second column represents the recording method MEG for each frequency band separately. Third column represents the combined approach (EEG+MEG). The numbers indicate the order of sources found for each frequency band separately.

The grand average results of all subjects for the analysis of significant coherent sources at the frequencies analyzed are displayed for both EC and EO conditions (Figs 3 and 4, respectively). In these Figs, the first source in each frequency band represents the highest power source and the following sources represent the coherent sources with the first source as the reference. In all the healthy subjects, the network of sources were identified for each of the recording modalities separately and then combined. For EEG, MEG, and the combined approach, similar networks of sources were identified for both conditions. For EC condition the following pattern of power and coherence was shown (see Fig 3): Delta activity was associated with coherent sources in the premotor cortex (source 1, BA6), middle cingulate cortex (source 2, BA32) and dorso-lateral prefrontal cortex (source 3, BA46); theta activity was related to the sources in the middle cingulate cortex (source 1, BA32), parietal cortex and precuneus (source 2, BA39 and BA7), medial prefrontal cortex (source 3, BA9) and dorso-lateral prefrontal cortex (source 4, BA46); alpha activity was attributed to the sources in the parietal cortex and precuneus (source 1, BA39 and BA7), occipital cortex (source 2, BA17), dorso-lateral prefrontal cortex (source 3, BA46), motor cortex (source 4, BA4), and thalamus (source 5, BA23); beta activity correlated with sources in the occipital cortex (source 1, BA17), parietal cortex and precuneus (source 2, BA39 and BA7), premotor cortex (source 3, BA6), dorso-lateral prefrontal cortex (source 4, BA46), inferior frontal gyrus (source 5, BA44), as well as thalamus (source 6); and gamma activity correlated with sources (S7 Fig) in the dorso-lateral prefrontal cortex (source 1, BA46), motor cortex (source 4, BA4), middle cingulate cortex (source 2, BA32) and thalamus (source 5, BA23).

For EO condition the neural oscillations were related to the following sources (see Fig 4): delta activity was associated with sources in the premotor cortex (source 1, BA6), parietal cortex (source 2, BA39), insula (source 3, BA16), and caudate nuclei (source 4); theta activity was related to sources in the medial and lateral prefrontal cortex (source 1, BA9 and BA10), inferior frontal gyrus (source 2, BA44), as well as orbitofrontal cortex (source 3, BA11); alpha activity was attributed to sources in the occipital cortex (source 1, BA17), middle cingulate cortex (source 2, BA32), medial prefrontal cortex (source 3, BA9), dorso-lateral prefrontal cortex (source 4, BA46), and thalamus (source 5); beta activity correlated with sources in the middle and posterior cingulate cortex (source 1, BA30 and BA31), premotor cortex (source 2, BA6), medial and dorso-lateral prefrontal cortex (source 3, BA9 and BA46) and thalamus (source 4); and gamma activity correlated with sources (S7 Fig) in the premotor cortex (source 2, BA6), occipital cortex (source 1, BA17), dorso-lateral prefrontal cortex (source 3, BA46) and parietal cortex (source 2, BA39).

No significant difference was found for the strength of coherence between conditions EO and EC (F_2,58_ = 1,86; p = 0,73). In case of the total interaction strength, EEG showed significantly (EEG vs. MEG—F_2,56_ = 26,43; p = 0,002; EEG vs. EEG+MEG—F_2,56_ = 28,21; p = 0,004; MEG vs. EEG+MEG–F_2,56_ = 29,16; p = 0,005) higher mean coherence values in all frequency bands compared to MEG and the combined EEG+MEG approach (Tables 1 and 2). The combined approach showed higher significant mean coherence values to the MEG alone.

**Table 1:** The mean and standard deviation of coherence and RPDC values between all the sources from all the subjects (separately for each recording method) for the condition EC.

Coherence (mean)	EEG	MEG	COMB (EEG+MEG)
Delta	0.31±0.05	0.19±0.04	0.28±0.04
Theta	0.26±0.03	0.15±0.03	0.24±0.02
Alpha	0.25±0.06	0.17±0.02	0.21±0.02
Beta	0.21±0.03	0.14±0.06	0.18±0.03
Gamma	0.24±0.04	0.18±0.05	0.21±0.02
RPDC (mean)	EEG	MEG	COMB (EEG+MEG)
Delta	0.18±0.02	0.12±0.04	0.23±0.04
Theta	0.15±0.03	0.10±0.03	0.20±0.02
Alpha	0.15±0.05	0.13±0.02	0.20±0.03
Beta	0.12±0.04	0.10±0.02	0.17±0.02
Gamma	0.12±0.03	0.10±0.03	0.20±0.02

**Table 2:** The mean coherence values and RPDC values between all the sources from all the subjects (separately for each recording method) for the condition EO.

Coherence (mean)	EEG	MEG	COMB (EEG+MEG)
Delta	0.25±0.04	0.17±0.05	0.21±0.03
Theta	0.19±0.03	0.12±0.04	0.17±0.05
Alpha	0.21±0.02	0.15±0.03	0.18±0.03
Beta	0.18±0.02	0.11±0.03	0.14±0.02
Gamma	0.17±0.03	0.10±0.02	0.13±0.02
RPDC (mean)	EEG	MEG	COMB (EEG+MEG)
Delta	0.18±0.04	0.14±0.04	0.22±0.02
Theta	0.20±0.02	0.16±0.04	0.26±0.02
Alpha	0.14±0.03	0.10±0.03	0.18±0.05
Beta	0.17±0.02	0.14±0.02	0.22±0.04
Gamma	0.16±0.03	0.13±0.03	0.20±0.03

In within subject statistics, for EO and EC conditions there were significant differences (F_2,56_ = 32,68; p = 0,001) for all the sources in all the frequency bands. However, for the recording modalities and for EO and EC conditions, there were no significant differences for both the maximum power (S5 Table) and coherence source (S6 Table) in all the frequency bands. We repeated the source analysis for all the 29 subjects for only the alpha frequency band by taking 64 EEG channels and 64 MEG sensors into consideration. We found the same network of cortical and sub-cortical sources for both the recording modalities.

### 3.3 Directional interactions between the networks of sources

**Fig 5:**
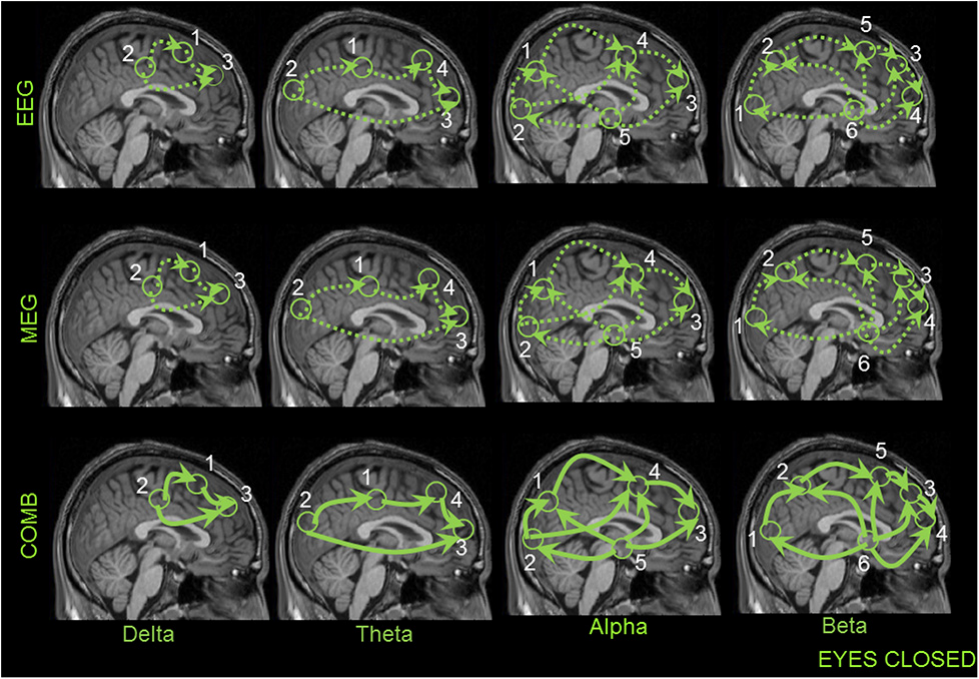
This figure illustrates the information flow between the coherent sources in the brain for the EC condition using EEG (first row), MEG (second row) and COMB (EEG+MEG) (Third row). The numbering of the sources are the same as in the previous Figs 3 and 4. The dotted lines indicate weaker interactions found between the sources for the recording methods EEG and MEG separately. The bold line with the arrow heads indicates significant higher directional interaction between the sources for only the combined approach (EEG+MEG).

**Fig 6:**
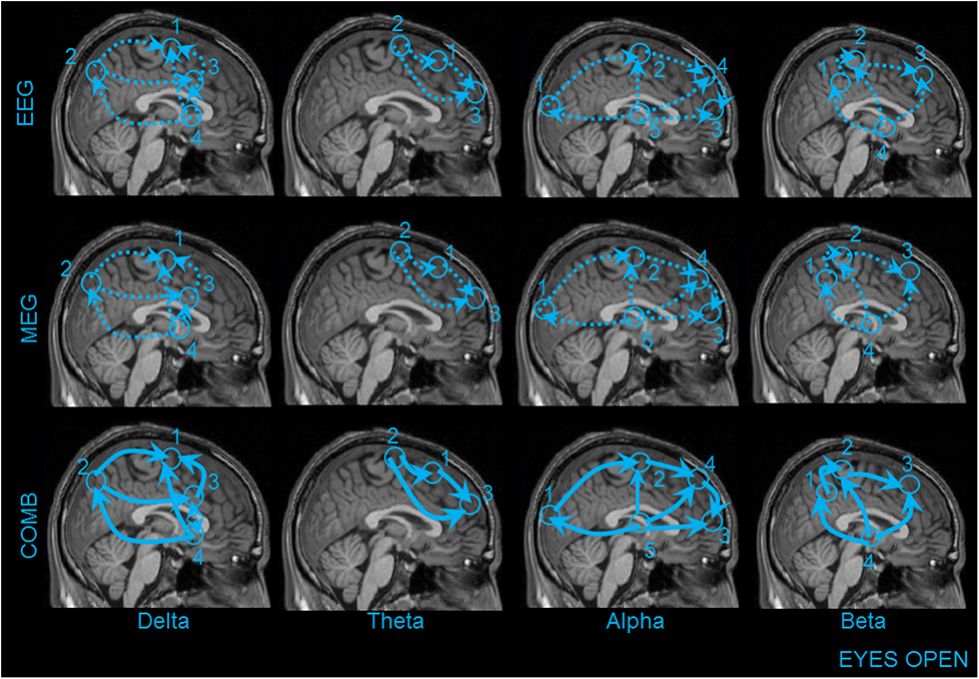
This figure illustrates the information flow between the coherent sources in the brain for the EC condition using EEG (first row), MEG (second row) and COMB (EEG+MEG) (Third row). The numbering of the sources are the same as in the previous Figs 3 and 4. The dotted lines indicate weaker interactions found between the sources for the recording methods EEG and MEG separately. The bold line with the arrow heads indicates significant higher directional interaction between the sources for only the combined approach (EEG+MEG).

The information flow between sources (same naming as in Fig 3) of brain activity for each frequency analysed is illustrated for both EC and EO data (Figs 5 and 6, respectively). In the following paragraph we will discuss only differences in the RPDC between the recording modalities.

During both the EC resting state and the EO condition, the direction of information flow for EEG, MEG and combined approach was not different between the sources for all the frequency bands. However, the combined approach showed significantly stronger connections in comparison to either of the modalities alone (indicated as bold lines: Figs 5 and 6) in each of the frequency bands. The mean RPDC values for the different frequency bands and for each of the modalities are shown in Table 1 for EC and Table 2 for EO condition. Finally, there was a significant difference between the recording modalities in the strength of direction of information flow (S7 Table). The TRT analyses emphasized the robustness of the above-mentioned results, as any significant causal interaction identified by RDPC were identified as strong (but not weak) asymmetry by the TRT. By using the simulation we were able to show that the RPR plays an important role for the increase in connection strength in the RPDC analysis for the combined approach (S3 and S4 Figs).

### 3.4 Relative-power-ratio ratio (RPR) and spatial resolution

The scalp level relative RPR on the selected electrodes/sensors showed significant difference between the two recording modalities. In all the five frequency bands the pattern remained similar (EEG>MEG—F_2,56_ = 41,06; p = 0,002). The source level RPR also indicated the same pattern for each frequency band and for each source significant difference between the recording modalities (EEG+MEG>EEG—F_2,56_ = 28,64; p = 0,006; EEG+MEG>MEG—F_2,56_ = 38,47; p = 0,002; EEG>MEG—F_2,56_ = 27,46; p = 0,002). The source level RPR showed that the combined approach had a significantly higher RPR compared to each of the recording modalities alone. EEG measurements showed higher scalp and source level RPR in comparison to the MEG alone. We were able to validate the results on the scalp and source level for both EEG and MEG recording methods and the simulation is depicted in S5 Fig In EEG and MEG recording modalities both the cortical and sub-cortical sources showed no significant difference for the number of voxels activated (S8 Table). However, the combined approach (EEG+MEG) had a significantly lower number of voxels activated for both cortical and sub-cortical sources in all frequency bands (S8 Table). By using the simulation we were able to show that the RPR plays an important role for the generation of focal source maps and also increases the coherence strength in the source analysis for the combined approach (S2 Fig).

The spectral and source level RPR showed the same trend of EEG > MEG (F_2,58_ = 26.63; p = 0.004) between the two recording modalities (S4 Fig). However, the spatial resolution got worse for MEG in comparison to all the 275 sensors but did not reach significance.

## Discussion

The main findings of the present study are: In both EEG and MEG recording modalities, all the analysed bands showed significant higher source and spectral absolute mean power for the EC compared to the EO condition. Comparative analysis of the spectral absolute mean power between the different recording modalities found EEG to be significantly higher than MEG across all frequency bands for the EC condition. The same trend was observed across all frequency bands, with the exception of the beta and gamma band, for the EO condition. For the source absolute mean power the combined approach had significantly higher power compared to both EEG and MEG. Between EEG and MEG, EEG showed significantly higher power than MEG in all the frequency bands, with the exception of the beta and gamma band, for both EC and EO conditions. In respect to the total interaction strength, EEG showed significantly higher mean coherence values in all frequency bands compared to the other two recording modalities MEG and the combined EEG+MEG approach. In the within subject statistics for between conditions EO and EC there were significant differences for all the sources in all the frequency bands. However, for the between recording modalities test there was no significant differences for the maximum power and coherence source in all the frequency bands.

During EO and EC resting state, the direction of information flow for EEG, MEG and the combined approach was no difference between the sources for all the frequency bands. However, the combined approach showed significantly stronger connections in each of the frequency bands. Finally, there was a significant difference between the recording modalities in the directionality for all frequency bands and sources, irrespective of the resting state. The RPDC values in all the five frequency bands showed a similar pattern (EEG+MEG>EEG>MEG) for both the conditions.

The scalp level RPR on the selected electrodes/sensors showed significant differences between the three recording modalities. In all the five frequency bands the pattern remained similar for both conditions (EEG+MEG > EEG > MEG) for this task. The source level RPR also indicated the same pattern for each frequency band and for each source with significant differences between the recording modalities for both conditions (EEG+MEG > EEG > MEG). Both, the scalp and source level RPR showed that the combined approach had a significantly higher RPR compared to the individual recording modalities. While EEG showed higher scalp and source level RPR in comparison to the MEG alone. For both EEG and MEG recording modalities the cortical and sub-cortical sources showed no significant difference in the number of activated voxels. However, the combined approach (EEG+MEG) had a significantly lower number of activated voxels for both cortical and sub-cortical sources in all the frequency bands.

### 4.1 Differences in absolute mean power and functional correlation

At the spectral level, the absolute mean values from delta, theta and alpha frequency bands was significantly higher for EEG compared to MEG in both conditions while the beta and gamma frequency was only significant in the EC condition. Comparisons of both conditions at the spectral level have been extensively studied using both EEG and MEG separately [16, 17]. The previous studies have demonstrated a greater power for EC condition compared with EO condition for all five frequency bands. In this study we had the advantage of comparing the absolute mean power at the source level across all frequency bands, under two conditions (EC and EO), in both EEG and MEG modality, as well as in a combined EEG+MEG modality. Except for the beta and gamma frequency, the trend (EEG+MEG>EEG>MEG) was similar in all other recorded frequency bands. The above discussed results were confirmed by using individual (i.e., IAF-based) band definitions rather than fixed pre-defined frequency bands [55]. Similar results were described in our previous study, which compared the spectral power between children and adults using EEG [18], as well as in previous studies utilizing MEG [58, 59]. Several EEG-fMRI studies have also shown absolute mean power differences between both EC and EO conditions during the resting state[60, 61]. These previous findings provide a strong basis for our study and indicate that the observed differences are not specific to the recording method used. In addition, these differences between the EC and EO conditions are also shown in studies using only fMRI [21, 62, 63].

The network of coherent sources for both the EC and EO conditions showed similar networks for both the recording modalities separately and combined. These networks were also found in our previous study using EEG alone in adults [18] and are also described in an earlier MEG study [64] and several fMRI resting state studies, with or without EEG [65, 66]. The functional correlation or coherence in both EC and EO conditions has been studied in either EEG [67–69] or MEG [70, 71] recording modalities, although primarily at the spectral level. In our previous study we identified differences in the coherence between children and adults in EEG [18] in both conditions. But, in this study we were able to compare the coherence strength between the recording modalities at the source level. The results showed the coherence strength with this trend (EEG>EEG+MEG>MEG) in all the frequency bands and in both conditions. The differences in trend for the recording modalities in power and coherence can be discussed from a recent model, which proposed that the decline in power and increase in coherence are complementary processes that support cognitive gains in the brain [72]. The changes in coherence in relation to power, namely between the recording modalities EEG and MEG, are also discussed in this review for resting state [73] and also other tasks in scalp and at the source level [1, 15]. The above studies indicate that the differences in the power and coherence are possible between the recording modalities. As the combined approach had higher mean source power, but low coherence because of the MEG, the combined recording method has a reduction in the strength of coherence. We hypothesize the differences we see in power and coherence between the recording modalities EEG and MEG are mainly due to these reasons. In respect to power, EEG captures the majority of the radial electric field, which are three times stronger for cortically spread sources, that is present in the resting state task. This is in comparison to the tangential magnetic field, which is weaker, and mainly captured by MEG[2, 74]. For coherence, the resting state involves long range connectivity spanning the whole brain, which is better observed in EEG compared to MEG, and focal cortical dynamics, which can be more clearly observed by MEG [74]. The effects of volume conduction, electrodes and sensors distance, spread and focal sources have been extensively tested with simulations and real data, and it is described that in certain brain states, EEG could dominate MEG in terms of power and coherence[75–77]. One more important point is the local magnetic fields which have the origin in sub-cortical regions may be distorted this could in turn have effects on the spatial accuracy in MEG[73]. The point spread function of the MEG strictly depends on the distance of the sensors from the sources which is always larger compared to the EEG[74]. To emphasize the common question of whether MEG is superior or inferior to EEG could be a question in the wrong direction. The right question to answer is whether MEG can add substantial information to the EEG. In this study and this particular task with the estimated parameters showed that the EEG could perform better to MEG.

### 4.2 Differences in effective connectivity

The most prominent finding of this study is that the combined approach showed stronger interactions to that of using the recording modalities separately, in each of the frequency bands for both EC and EO states. There was no significant difference in the directions of information flow between the sources for both EEG and MEG in all the frequency bands. These results were also confirmed by the time reversal technique, which showed that in the case of EEG there was no zero lagged coherences (weak symmetries) detected but only strong symmetries [18, 50]. The connections which are exhibited in all the three recording modalities EEG, MEG and EEG+MEG will not be discussed in detail, because all these connections are well described in earlier resting state studies using either of these recording modalities [18, 78, 79], or fMRI[60, 80]. However, in this study we can confirm that the causalities found are similar in the two different recording modalities measured. In addition, when analyzed together they provide us the surety of the identified effective connectivity within the network of sources with an independent recording method. But, the between recording modalities statistical test with the reference voxel showed the same trend (EEG+MEG>EEG>MEG) as in the mean source power over all the recording modalities. This in turn indicates that the causality benefits from the combined approach more so than in each of the recording method alone.

### 4.3 Differences in RPR and spatial resolution

The scalp RPR followed the same trend EEG>MEG as in power, coherence and directionality between the recording modalities when analyzed separately. This is not only true for this study but is also shown in earlier studies with epileptic focal or distributed sources and simulation studies [30, 31, 74]. EEG is better at capturing the distributed sources in the resting state than focal cortical sources with fewer electrodes compared to MEG on the scalp [8, 81]. The same holds true for the source level RPR, that EEG has a better RPR than MEG which is dependent on a task that induces sources which are spread over the cortex and not focal [82]. The combined approach (EEG+MEG) had the best RPR at the source level compared to both the recording modalities alone. The advantages of combining them to get the best possible RPR for source localization algorithms [7, 9, 13] has not only been proven in epilepsy [83], but also in cognitive tasks [84].

The spatial resolution showed that between EEG and MEG there was no significant difference in the number of activated voxels when analyzed separately. The number of electrodes or sensors is not the important factor but the RPR plays the major role in the accuracy of location of sources both in cortical [6, 8, 11] and sub-cortical regions [10, 82]. However, the combined approach showed a better spatial resolution in comparison to the individual recording modalities indicating that both these recording modalities give complimentary information which is vital in the accuracy of identifying the sources. The combined approach can be used to test and compare both the recording modalities alone. This will help in the selection of a recording method for a specific task, and will be easier for the research groups who have limited access to simultaneous acquisition systems.

### 4.4 Limitations of the study

This study has several limitations which need to take into consideration while interpreting the results of this study. Firstly, all the comparisons are performed with a 64-channel EEG system and a 275 sensor MEG system. In order to circumvent this discrepancy, and ensure the observed differences are not merely due to the number of electrodes/sensors used [8, 85], we repeated the source analysis for all the 29 subjects for only the alpha frequency band by taking 64 EEG channels and 64 MEG sensors into consideration. We found the same network of cortical and sub-cortical sources for both the recording modalities. The spectral and source level RPR showed the same trend of EEG > MEG (F_2,58_ = 26.63; p = 0.004) between the two recording modalities (S4 Fig). However, the spatial resolution got worse for MEG in comparison to all the 275 sensors but did not reach significance. Secondly, in this study no realistic head model was used. Only semi-realistic head models with individual electrodes and sensors locations were applied. The advantages of using individual MRI for boundary element or finite element methods in localizing electrical sources are shown in previous studies [86, 87]. The realistic head model will possibly increase the localization accuracy for EEG. The head modelling makes adverse effects on the interpretation of the results on the individual subject level. However, we want to point out that by taking the combined approach for the forward modelling, and also standard isotopic conductivity values [88], we are comparing the group differences and not claiming any individual subject’s results. The first inconsistency of the number of MEG sensors and the number of EEG channels in our data was tested only for the alpha frequency. The results from the other frequency bands should be interpreted with care and in future studies to have equal number of sensors would be ideal. The second inconsistency of the semi-realistic model for EEG should be dealt with care even though we discuss only group differences and not at individual subject level.

Finally, this study reveals sub-cortical sources using DICS (i.e., the thalamus). The identification of deep regions in the brain with scalp recordings is still under debate. Several studies using MEG [22, 27, 89] and EEG [24–26, 90] have shown that this beam former approach is a powerful tool for locating deep sub-cortical sources[15, 18, 91].Furthermore, it is worthy to mention that these results should be further tested with other multi-beam former analysis [92] and alternative comparative source analysis algorithms which have been applied to low RPR signals and proven to be better than DICS [93].

## Conclusion

In this study we demonstrate that the combined approach (EEG+MEG) has several advantages over each of the recording modalities alone for this specific resting state task. The outcome of this study could be used as an initial standardization procedure for future clinical studies in this research area; depending on both the recording tools and methods available and the questions to be answered. All the differences in power, causality, RPR and spatial resolution lead to the conclusion that the combined EEG+MEG approach is better than EEG or MEG alone and that EEG outperforms MEG. For the parameter coherence EEG outperforms both the combined EEG+MEG approach and MEG alone.

## Supporting Information

S1 Fig Shows the 2D representation of the simulated five dipoles for each condition eyes closed (EC) (first two columns), eyes open (EO) (third and fourth column) and for all the three recording methods EEG (first row), MEG (second row) separately and the combined approach (third row). The left hemisphere is shown in first and third column and the right hemisphere is shown in the second and the fourth column.
(TIF)

S2 Fig Shows the simulations results of the source analysis for each condition eyes closed (EC) (first three columns), eyes open (EO) (second three columns) and for the alpha frequency band separately for each recording method. First column represents the recording method EEG, second column represents the recording method MEG and the third column represents the combined approach (EEG+MEG). The numbers indicate the order of sources found for alpha frequency band.
(TIF)

S3 Fig This figure illustrates the results of the information flow from the simulation between the coherent sources in the brain for the EC condition using EEG (first row), MEG (second row) and COMB (EEG+MEG) (Third row). The numbering of the sources are the same as in the previous Figs 3 and 4. The dotted lines indicate weaker interactions found between the sources for the MEG and dashed line increased connectivity for the EEG modality. The bold line with the arrow heads indicates significant higher directional interaction between the sources for only the combined approach (EEG+MEG).
(TIF)

S4 Fig Simulation results showing the mean RPDC values for eyes open and eyes closed condition. MEG (black bars), EEG (white bars), EEG+MEG (grey bars). (TIF)

S5 Fig Results of the spectral and source RPR for. MEG (black bars), EEG (white bars). (TIF)

S6 Fig Results of the spectral and source absolute power of the frequency band gamma for eyes closed (EC) and eyes open (EO) condition. Mean band power (with standard deviation) is shown for EEG (black bars), MEG (white bars), and EEG+MEG (grey bars). Significant recording method differences are indicated by * (p < 0.05).
(TIF)

S7 Fig First column represents the recording method EEG for the frequency band gamma showing the grand average statistical map of network of sources for the eyes closed (EC) condition. Second column represents the recording method MEG for the gamma band separately. Third column represents the combined approach (EEG+MEG). The numbers indicate the order of sources found for gamma frequency band separately. Additionally, the figure illustrates the information flow between the coherent sources in the brain for the EC condition using EEG (first row), MEG (second row) and COMB (EEG+MEG) (Third row). The dotted lines indicate weaker interactions found between the sources for the recording methods EEG and MEG separately. The bold line with the arrow heads indicates significant higher directional interaction between the sources for only the combined approach (EEG+MEG).
(TIF)

S1 Table The relative-power-ratio (RPR) values for all the sources separately for each recording method for the condition EC and EO. (DOCX)

S2 Table The individual alpha frequencies (IAF) t-values and p-values for each frequency band separately. (DOCX)

S3 Table The t and p values of the statistics on the spectral absolute power for Eyes closed/Eyes open condition (EEG Vs. MEG) for each frequency band separately. (DOCX)

S4 Table The t and p values of the statistics on the source absolute power for Eyes closed/Eyes open condition for each frequency band separately. (DOCX)

S5 Table The t and p values of the statistics on the maximum source power for Eyes closed/Eyes open condition for each frequency band separately. (DOCX)

S6 Table The t and p values of the statistics on the maximum source coherence for Eyes closed/Eyes open condition for each frequency band separately. (DOCX)

S7 Table The t and p values of the statistics on the RPDC strength for Eyes closed/Eyes open condition for each frequency band separately. (DOCX)

S8 Table The t and p values of the statistics on the number of activated voxels for Eyes closed/Eyes open condition for each frequency band separately. (DOCX)
