# Supplementary figures and images for: EEG-MEG Integration Enhances the Characterization of Functional and Effective Connectivity in the Resting State Network
Source: PLoS One. 2015 Oct 28;10(10):e0140832. doi: 10.1371/journal.pone.0140832 (PMC4624977; doi:10.1371/journal.pone.0140832)

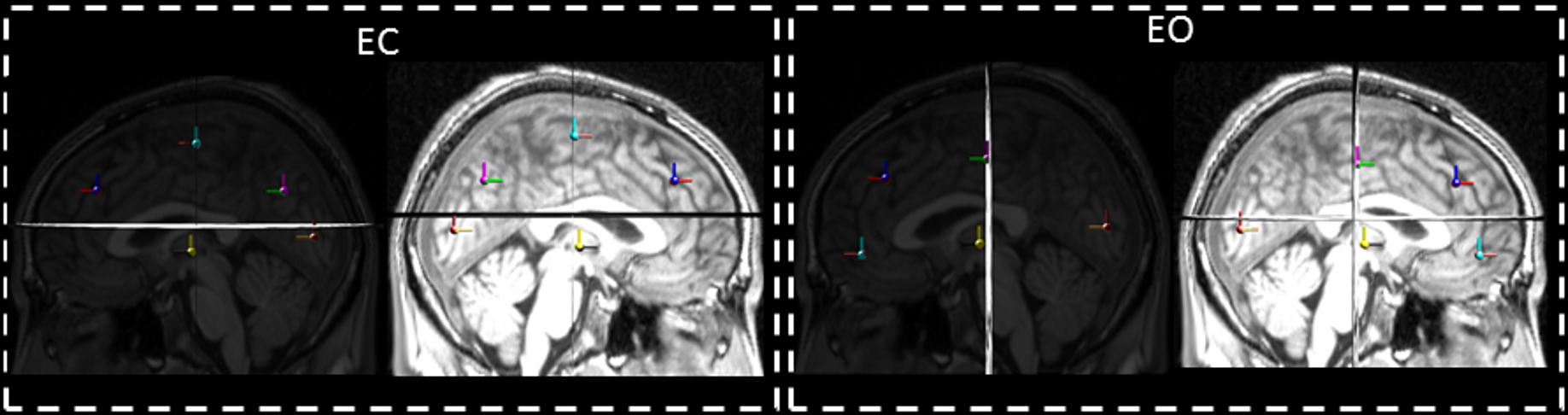

Supplement: S1 Fig — The left hemisphere is shown in first and third column and the right hemisphere is shown in the second and the fourth column. (TIF) [file pone.0140832.s001.tif]

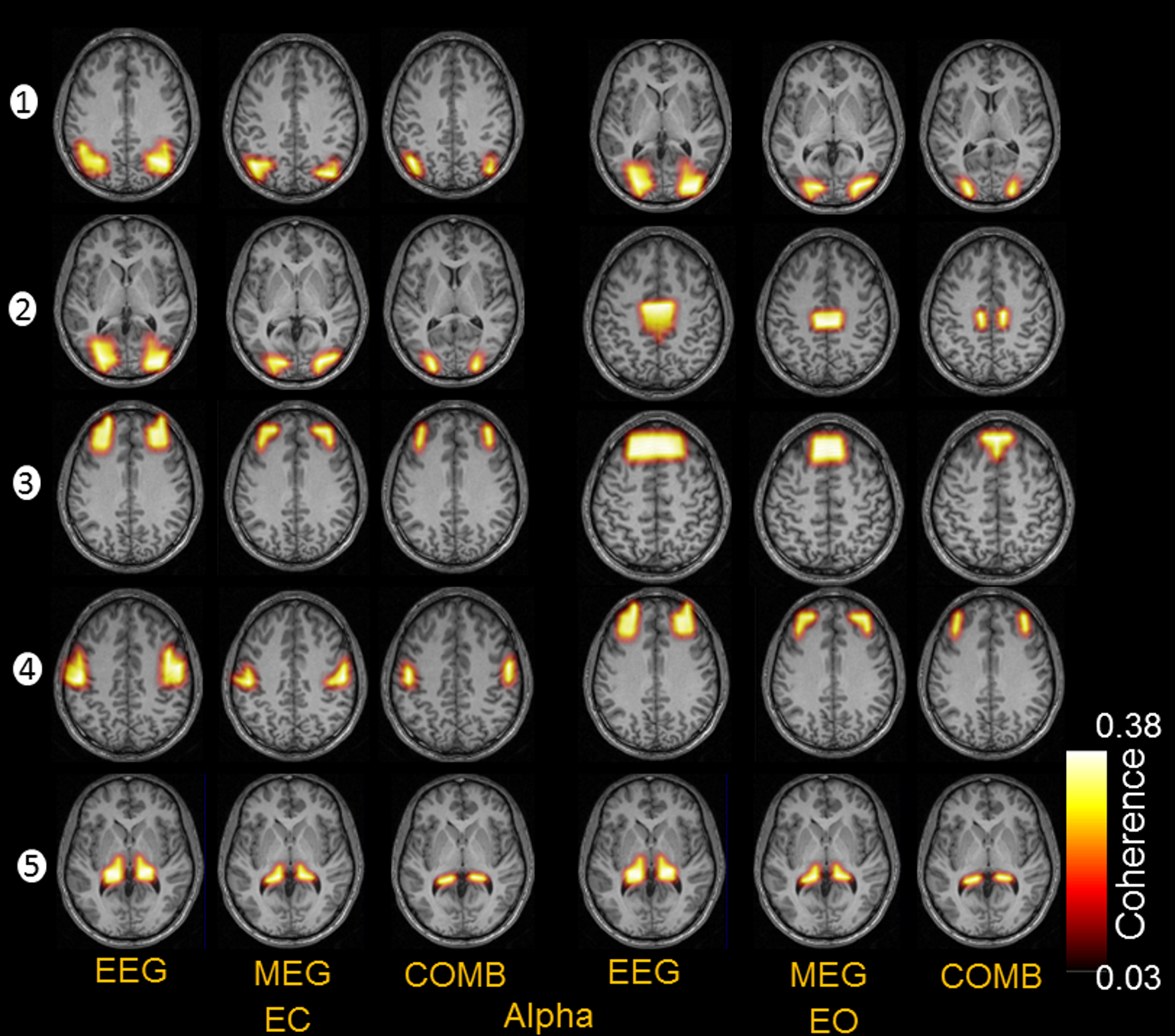

Supplement: S2 Fig — First column represents the recording method EEG, second column represents the recording method MEG and the third column represents the combined approach (EEG+MEG). The numbers indicate the order of sources found for alpha frequency band. (TIF) [file pone.0140832.s002.TIF]

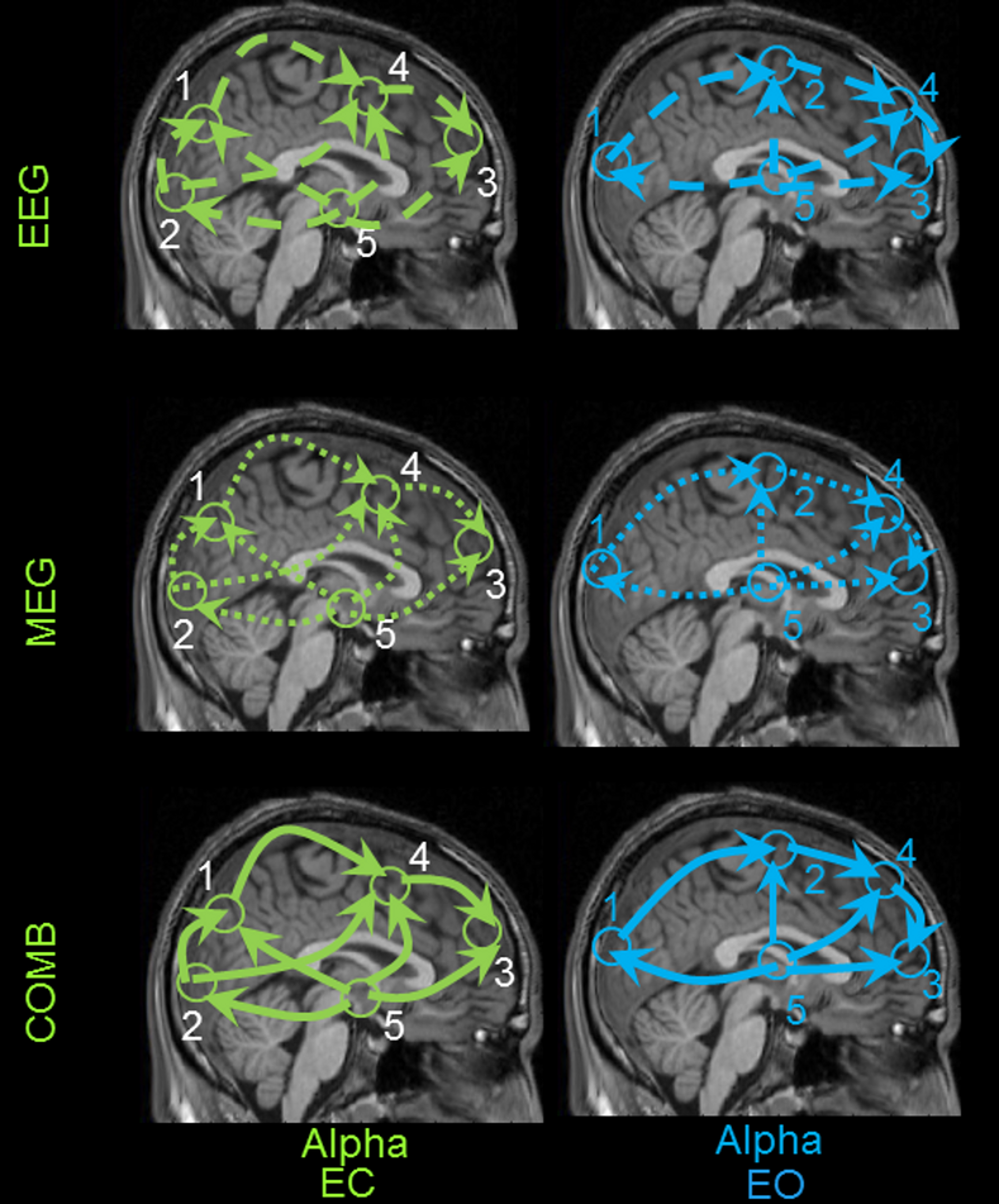

Supplement: S3 Fig — The numbering of the sources are the same as in the previous Figs 3 and 4. The dotted lines indicate weaker interactions found between the sources for the MEG and dashed line increased connectivity for the EEG modality. The bold line with the arrow heads indicates significant higher directional interaction between the sources for only the combined approach (EEG+MEG). (TIF) [file pone.0140832.s003.TIF]

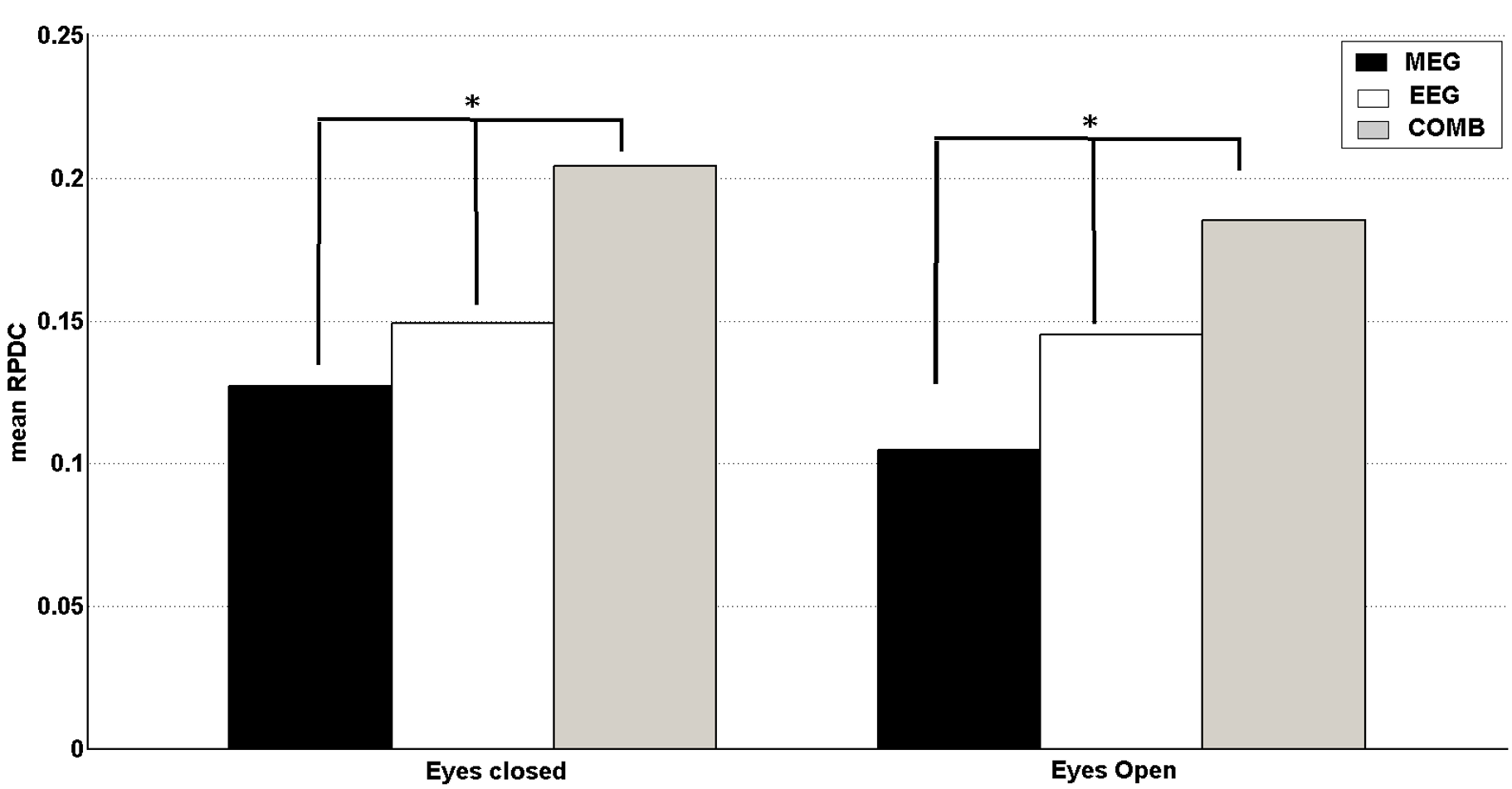

Supplement: S4 Fig — (TIF) [file pone.0140832.s004.TIF]

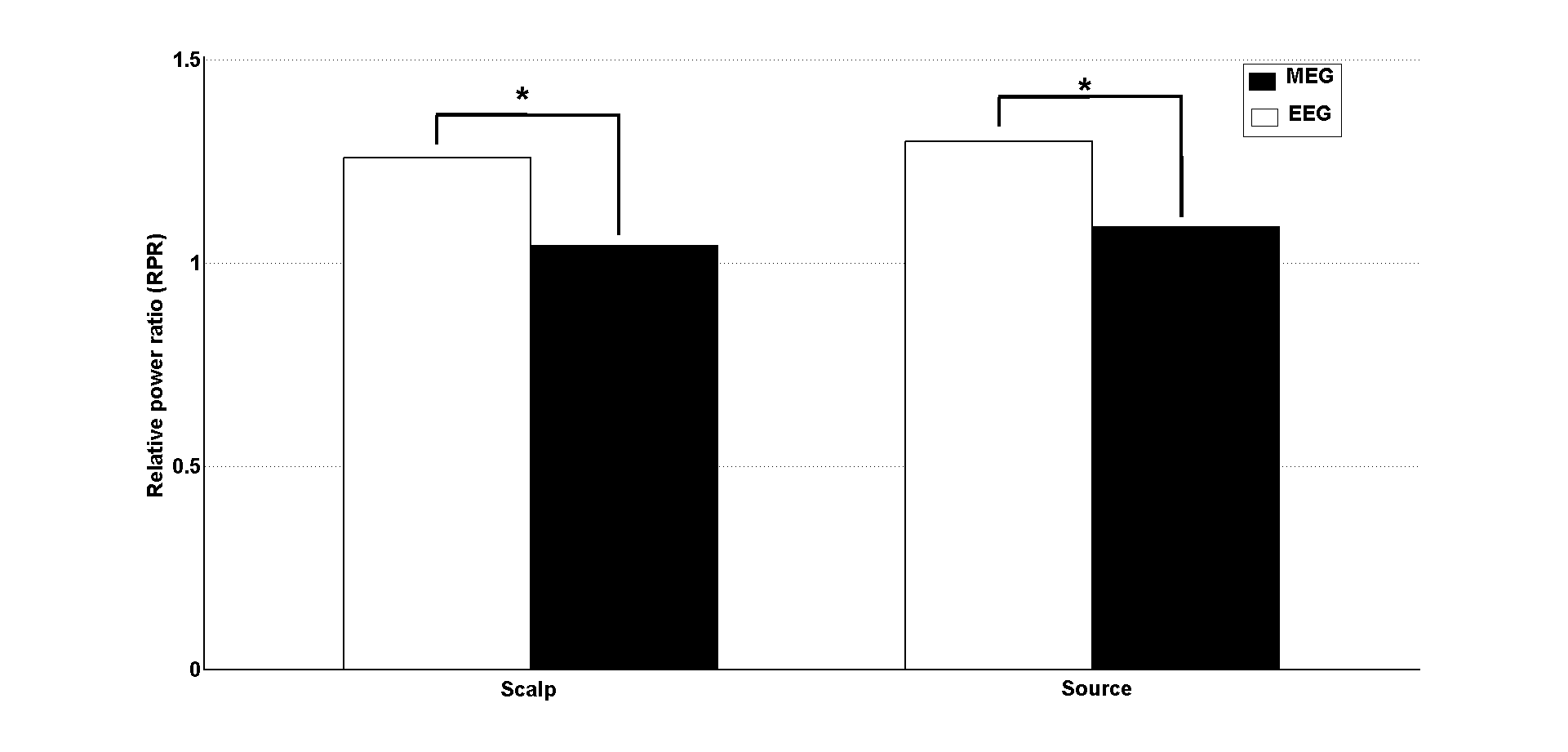

Supplement: S5 Fig — (TIF) [file pone.0140832.s005.tif]

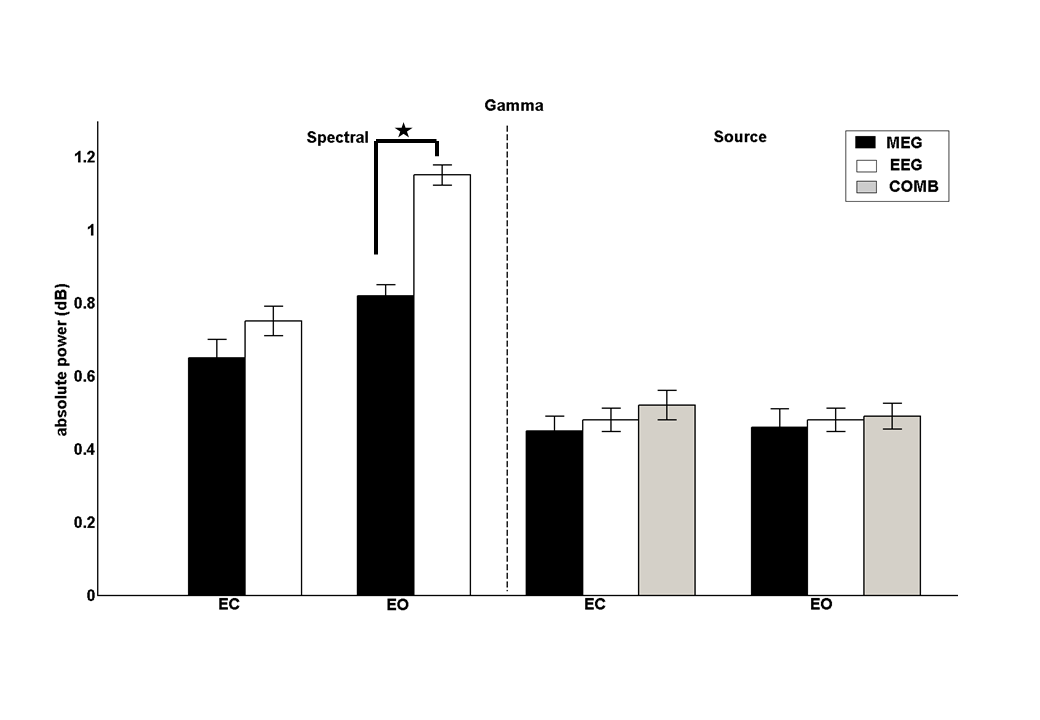

Supplement: S6 Fig — Mean band power (with standard deviation) is shown for EEG (black bars), MEG (white bars), and EEG+MEG (grey bars). Significant recording method differences are indicated by * (p < 0.05). (TIF) [file pone.0140832.s006.tif]

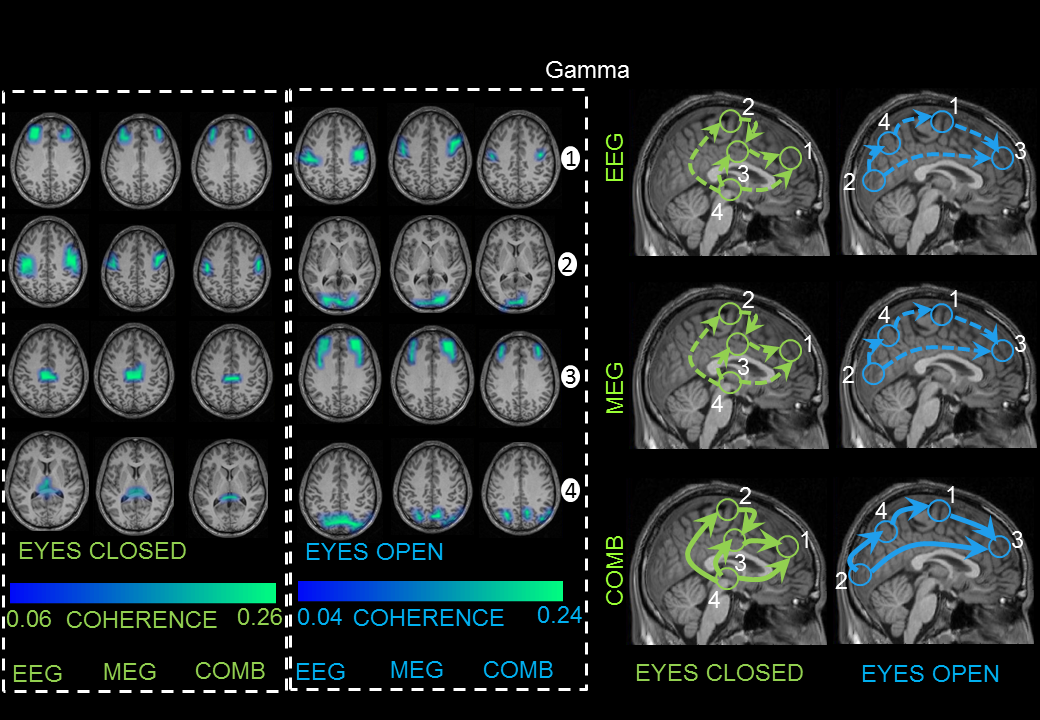

Supplement: S7 Fig — Second column represents the recording method MEG for the gamma band separately. Third column represents the combined approach (EEG+MEG). The numbers indicate the order of sources found for gamma frequency band separately. Additionally, the figure illustrates the information flow between the coherent sources in the brain for the EC condition using EEG (first row), MEG (second row) and COMB (EEG+MEG) (Third row). The dotted lines indicate weaker interactions found between the sources for the recording methods EEG and MEG separately. The bold line with the arrow heads indicates significant higher directional interaction between the sources for only the combined approach (EEG+MEG). (TIF) [file pone.0140832.s007.tif]
