# Supplementary material for: EEG-MEG Integration Enhances the Characterization of Functional and Effective Connectivity in the Resting State Network
Source: PLoS One. 2015 Oct 28;10(10):e0140832. doi: 10.1371/journal.pone.0140832 (PMC4624977; doi:10.1371/journal.pone.0140832)
Supplement: S1 Table — (DOCX) [file pone.0140832.s008.docx]

**S1 Table:**

| RPR | First source | Second source | Third source | Fourth source | Fifth source |
| --- | --- | --- | --- | --- | --- |
| EEG | 0.8 | 0.7 | 0.6 | 0.5 | 0.3 |
| MEG | 0.7 | 0.6 | 0.4 | 0.3 | 0.2 |
| COMB | 1.4 | 1.2 | 1.0 | 0.8 | 0.6 |
