# Supplementary material for: EEG-MEG Integration Enhances the Characterization of Functional and Effective Connectivity in the Resting State Network
Source: PLoS One. 2015 Oct 28;10(10):e0140832. doi: 10.1371/journal.pone.0140832 (PMC4624977; doi:10.1371/journal.pone.0140832)
Supplement: S2 Table — (DOCX) [file pone.0140832.s009.docx]

**S2 Table:**

| Bands | mean±std | t(29) | p-values |
| --- | --- | --- | --- |
| Delta | 2.1±1.2 | 0.76 | 0.0824 |
| Theta | 5.4±2.1 | 0.54 | 0.1243 |
| Alpha | 10.4±2.3 | 0.86 | 0.0765 |
| Beta | 23.2±6.8 | 0.46 | 0.1655 |
| Gamma | 41.2±6.4 | 0.35 | 0.2136 |
