# Supplementary material for: EEG-MEG Integration Enhances the Characterization of Functional and Effective Connectivity in the Resting State Network
Source: PLoS One. 2015 Oct 28;10(10):e0140832. doi: 10.1371/journal.pone.0140832 (PMC4624977; doi:10.1371/journal.pone.0140832)
Supplement: S3 Table — (DOCX) [file pone.0140832.s010.docx]

**S3 Table:**

| Bands | t(29) | p-values |
| --- | --- | --- |
| Delta | 2.79/2.76 | 0.0025/0.0070 |
| Theta | 3.35/3.37 | 0.0005/0.0047 |
| Alpha | 3.67/2.58 | 0.0016/0.0007 |
| Beta | 0.52/3.67 | 0.1676/0.0058 |
| Gamma | 0.60/3.45 | 0.1230/0.0064 |
