# Supplementary material for: EEG-MEG Integration Enhances the Characterization of Functional and Effective Connectivity in the Resting State Network
Source: PLoS One. 2015 Oct 28;10(10):e0140832. doi: 10.1371/journal.pone.0140832 (PMC4624977; doi:10.1371/journal.pone.0140832)
Supplement: S4 Table — (DOCX) [file pone.0140832.s011.docx]

**S4 Table:**

| Bands | EEG Vs MEG | EEG Vs  EEG+MEG | MEG Vs EEG+MEG | EEG Vs MEG | EEG Vs  EEG+MEG | | MEG Vs EEG+MEG |
| --- | --- | --- | --- | --- | --- | --- | --- |
| Delta | 2.57/2.67 | 3.09/3.32 | 3.76/2.91 | 0.007/0.006 | 0.003/0.004 | | 0.003/0.003 |
| Theta | 2.60/3.22 | 3.65/3.15 | 2.83/2.57 | 0.002/0.004 | | 0.002/0.006 | 0.006/0.006 |
| Alpha | 2.50/3.55 | 2.80/3.77 | 3.68/2.59 | 0.007/0.005 | | 0.004/0.005 | 0.005/0.001 |
| Beta | 0.81/0.76 | 0.95/0.51 | 1.06/0.63 | 0.076/0.119 | | 0.082/0.183 | 0.125/0.175 |
| Gamma | 0.70/0.60 | 1.06/0.61 | 1.02/0.57 | 0.093/0.069 | | 0.091/0.151 | 0.214/0.148 |
