# Supplementary material for: EEG-MEG Integration Enhances the Characterization of Functional and Effective Connectivity in the Resting State Network
Source: PLoS One. 2015 Oct 28;10(10):e0140832. doi: 10.1371/journal.pone.0140832 (PMC4624977; doi:10.1371/journal.pone.0140832)
Supplement: S5 Table — (DOCX) [file pone.0140832.s012.docx]

**S5 Table:**

| Bands | EEG Vs MEG | EEG Vs  EEG+MEG | MEG Vs EEG+MEG | EEG Vs MEG | EEG Vs  EEG+MEG | | MEG Vs EEG+MEG |
| --- | --- | --- | --- | --- | --- | --- | --- |
| Delta | 0.41/0.68 | 0.42/0.55 | 0.78/0.46 | 0.197/0.197 | 0.072/0.130 | | 0.170/0.173 |
| Theta | 0.60/0.55 | 0.71/0.47 | 0.51/0.67 | 0.087/0.143 | | 0.126/0.107 | 0.177/0.120 |
| Alpha | 0.43/0.54 | 0.76/0.53 | 0.46/0.60 | 0.159/0.124 | | 0.096/0.050 | 0.195/0.176 |
| Beta | 0.49/0.48 | 0.53/0.48 | 0.69/0.77 | 0.177/0.078 | | 0.172/0.145 | 0.061/0.085 |
| Gamma | 0.73/0.56 | 0.71/0.43 | 0.69/0.70 | 0.068/0.050 | | 0.117/0.086 | 0.172/0.110 |
