# Supplementary material for: EEG-MEG Integration Enhances the Characterization of Functional and Effective Connectivity in the Resting State Network
Source: PLoS One. 2015 Oct 28;10(10):e0140832. doi: 10.1371/journal.pone.0140832 (PMC4624977; doi:10.1371/journal.pone.0140832)
Supplement: S6 Table — (DOCX) [file pone.0140832.s013.docx]

**S6 Table:**

| Bands | EEG Vs MEG | EEG Vs  EEG+MEG | MEG Vs EEG+MEG | EEG Vs MEG | EEG Vs  EEG+MEG | | MEG Vs EEG+MEG |
| --- | --- | --- | --- | --- | --- | --- | --- |
| Delta | 0.56/0.50 | 0.68/0.79 | 0.79/0.44 | 0.119/0.192 | 0.133/0.060 | | 0.072/0.193 |
| Theta | 0.61/0.49 | 0.71/0.73 | 0.62/0.56 | 0.194/0.164 | | 0.060/0.075 | 0.129/0.061 |
| Alpha | 0.74/0.67 | 0.68/0.64 | 0.44/0.69 | 0.136/0.187 | | 0.192/0.171 | 0.096/0.184 |
| Beta | 0.73/0.72 | 0.70/0.60 | 0.65/0.71 | 0.124/0.074 | | 0.156/0.195 | 0.175/0.050 |
| Gamma | 0.59/0.52 | 0.75/0.43 | 0.63/0.72 | 0.098/0.094 | | 0.199/0.198 | 0.146/0.170 |
