# Supplementary material for: EEG-MEG Integration Enhances the Characterization of Functional and Effective Connectivity in the Resting State Network
Source: PLoS One. 2015 Oct 28;10(10):e0140832. doi: 10.1371/journal.pone.0140832 (PMC4624977; doi:10.1371/journal.pone.0140832)
Supplement: S7 Table — (DOCX) [file pone.0140832.s014.docx]

**S7 Table:**

| Bands | EEG Vs MEG | EEG Vs  EEG+MEG | MEG Vs EEG+MEG | EEG Vs MEG | EEG Vs  EEG+MEG | | MEG Vs EEG+MEG |
| --- | --- | --- | --- | --- | --- | --- | --- |
| Delta | 3.10/3.26 | 2.74/3.21 | 3.26/2.50 | 0.002/0.007 | 0.005/0.004 | | 0.003/0.004 |
| Theta | 3.22/3.38 | 3.71/2.47 | 2.50/2.59 | 0.007/0.002 | | 0.003/0.003 | 0.007/0.007 |
| Alpha | 2.44/3.14 | 2.48/2.43 | 3.50/2.53 | 0.006/0.007 | | 0.005/0.004 | 0.005/0.001 |
| Beta | 2.45/3.56 | 3.35/3.24 | 2.73/2.74 | 0.004/0.007 | | 0.003/0.007 | 0.005/0.000 |
| Gamma | 2.88/3.59 | 2.56/3.51 | 2.55/3.60 | 0.007/0.004 | | 0.006/0.006 | 0.003/0.005 |
