# Supplementary material for: EEG-MEG Integration Enhances the Characterization of Functional and Effective Connectivity in the Resting State Network
Source: PLoS One. 2015 Oct 28;10(10):e0140832. doi: 10.1371/journal.pone.0140832 (PMC4624977; doi:10.1371/journal.pone.0140832)
Supplement: S8 Table — (DOCX) [file pone.0140832.s015.docx]

**S8 Table:**

| Bands | EEG Vs MEG | EEG Vs  EEG+MEG | MEG Vs EEG+MEG | EEG Vs MEG | EEG Vs  EEG+MEG | | MEG Vs EEG+MEG |
| --- | --- | --- | --- | --- | --- | --- | --- |
| Delta | 0.63/0.78 | 3.38/3.43 | 3.76/2.57 | 0.086/0.059 | 0.007/0.006 | | 0.002/0.006 |
| Theta | 0.75/0.60 | 3.31/3.12 | 3.05/3.32 | 0.089/0.065 | | 0.003/0.006 | 0.007/0.007 |
| Alpha | 0.72/0.59 | 2.86/3.33 | 2.81/3.46 | 0.122/0.112 | | 0.003/0.007 | 0.004/0.008 |
| Beta | 0.73/0.53 | 2.56/2.61 | 3.18/3.00 | 0.107/0.183 | | 0.007/0.006 | 0.001/0.007 |
| Gamma | 0.79/0.51 | 2.43/3.75 | 2.77/3.46 | 0.113/0.092 | | 0.003/0.003 | 0.003/0.001 |
